# Occurrence and Risk Factors for Unplanned Central Venous Catheter Removal in Critically Ill Patients: A Multicenter Cohort Study

**DOI:** 10.1155/nrp/7640284

**Published:** 2025-09-04

**Authors:** Xiaofeng He, Chunlei Li, Zhe Wang, Mayi Yang, Tianjun Zhou, Ying Gu, Yuxia Zhang, Wenchao Wang, Wenyan Pan, Yan Hu

**Affiliations:** ^1^Fudan University School of Nursing, Shanghai, China; ^2^Fudan University Evidence-Based Nursing Centre, Shanghai, China; ^3^College of Nursing, Dali University, Dali, Yunnan, China; ^4^Intensive Care Unit, Fudan University Zhongshan Hospital, Shanghai, China; ^5^Operating Room, Fudan University Zhongshan Hospital, Shanghai, China; ^6^Operating Room, Fudan University Minhang Hospital, Shanghai, China; ^7^Nursing Department, Children's Hospital of Fudan University, Shanghai, China; ^8^Nursing Department, Fudan University Zhongshan Hospital, Shanghai, China; ^9^Emergency Department, Children's Hospital of Fudan University, Shanghai, China

**Keywords:** central venous catheter (CVC), intensive care unit, risk factor, unplanned removal

## Abstract

**Background:** Central venous catheters (CVCs) are crucial for critically ill patients but pose risks of complications and unplanned removal, which can interrupt treatment, prolong hospital stays, and increase mortality. This investigation sought to examine the occurrence and risk factors for unplanned CVC removal among intensive care patients in China.

**Methods:** A multicenter cohort study was conducted across 22 public tertiary hospitals throughout China, from September 4, 2023, to February 29, 2024, enrolling critically ill patients with CVCs. Cox proportional hazards regression models were used to assess the risk factors for unplanned CVC removal.

**Results:** The study comprised a total of 2680 first-time CVC insertion events (one per patient) in critically ill patients. 1151 (42.95%) CVCs were removed; most (*n* = 832, 31.04%) were elective. A total of 153 CVCs were removed prematurely (5.71%; 95% CI: 4.84–6.68), and infection-related complication was the leading cause (*n* = 124, 4.63%; 95% CI: 3.85–5.50; 5.26 per 1000 CVC days). Independent risk factors included male gender (HR, 2.04; 95% CI: 1.40–2.99; *p* < 0.001), neurological disorders (HR, 2.41; 95% CI: 1.50–3.86; *p* < 0.001), and mechanical ventilation (HR, 1.71; 95% CI: 1.09–2.70; *p*=0.02), while urgent insertion reduced the risk (HR, 0.52; 95% CI: 0.29–0.92; *p*=0.02). In subgroup analysis, diagnosis with neurological disorders (HR, 2.31; 95% CI 1.40–3.81, *p*=0.001), and urgent CVC insertion (HR, 0.41; 95% CI 0.21–0.82, *p*=0.01) were significantly associated with unplanned CVC removal in males but not in females (*p* > 0.05). No significant interactions were found between gender and diagnosis, mechanical ventilation, or urgent insertion (all *p* > 0.05).

**Conclusions:** Unplanned CVC removal occurred in 5.71% of cases, primarily due to infection. Identified risk factors (male gender, neurological disorders, and mechanical ventilation) and protective factors (urgent insertion) highlight targets for preventive strategies in critical care.

## 1. Introduction

The placement of central venous catheters (CVCs) is an essential procedure in the care of critically ill patients, serving multiple functions such as cardiovascular monitoring, rapid fluid resuscitation, medication/nutrition administration, and dialysis [1, 2]. CVCs are extensively utilized, with estimates of about 74.1 CVC days/100 patient days in Europe [3] and surpassing 150 million units in annual sales in the United States [4]. Although CVCs facilitate easy access to various treatments for critically ill patients, they also pose an elevated risk of catheter-related mechanical, infectious, thrombotic, or other complications, which lead to increased morbidity and mortality, as well as unplanned catheter removal [5, 6]. Studies suggest that around 3% of CVC placements result in severe complications, with a predicted rate of 30.2 (95% CI, 21.8–43.0) per 1000 patients catheterized for 3 days, potentially acquiring serious adverse events (arterial puncture, collapsed lung, sepsis, or blood clot) [6], and the rate of unplanned CVC removal for critically ill patients ranged from 1.5 to 15 per 1000 CVC days [7–9]. It is well established that CVC-related complications and unplanned CVC removal are associated with substantial clinical and economic consequences. These adverse events can lead to delayed treatment of the primary condition, prolonged hospitalization, increased healthcare costs, and elevated mortality rates, collectively imposing significant burdens on both patient outcomes and healthcare systems [10–12].

Unplanned CVC removal poses significant patient safety risks, with evidence suggesting most cases are preventable through targeted interventions [9, 13, 14]. Several studies have compared the occurrence of unplanned CVC removal, multiple factors have been proposed to be associated with unplanned CVC removal [7, 14, 15]. Curtis et al.'s multi-site cohort study showed that among the removed CVCs, nearly two in every five (42%) were removed prematurely, primarily due to infection (23%), with catheter type, urgency of the procedure, concurrent CVCs, and insertion technology influencing removal risk [14]. A long-term, prospective study by Garonzi et al. reported that about 20% of CVCs were prematurely removed, primarily due to mechanical complications, with age and the type of CVC as key predictors for early catheter removal [16]. A retrospective study conducted in North West England by Balmforth et al. highlighted additional risk factors, including suturing technique, delirium, the condition of lifting and handling the patient, and multi-lumen [9]. Current evidence demonstrates marked heterogeneity in risk factors for unplanned CVC removal, and most available data are derived from oncologic populations. Notably, risk factors specific to critically ill patients remain underreported in the literature. This multicenter cohort study was specifically designed to measure the occurrence of unplanned CVC removal for critically ill patients and identify risk factors through standardized prospective assessment, which could provide a basis for developing targeted preventive strategies.

## 2. Methods

### 2.1. Ethical Considerations

Ethical approval was obtained to conduct the study from the Institutional Review Board of Fudan University School of Nursing, China (approval no. IRB # 2023-4-10). Written informed consent was obtained from all participants or their legal guardians (including immediate family members, patient-designated guardians, or court-appointed guardians) prior to CVC placement or within 24 h of ICU admission. It guaranteed the anonymization and confidentiality of all participant data and explicitly addressed rights regarding data use for research and publication. Participants or their legal guardians were informed of their right to withdraw from the study at any time without any detriment to their clinical care. This study was conducted according to the guidelines of the Strengthening the Reporting of Observational Studies in Epidemiology (STROBE) [17] (Supporting Table S1).

### 2.2. Design and Setting

A prospective multicenter cohort study was carried out in 22 tertiary public hospitals spanning all major geographic regions of China (Eastern, Northeastern, Central, Southern, Southwestern, Northern, and Northwestern) from September 4, 2023, to February 29, 2024, enrolling critically ill patients with intensive care unit (ICU) stays > 48 h and indwelling CVCs for > 24 h, while excluding those transferred from external facilities with a pre-existing CVC or concurrent use of hemodialysis catheters, peripherally inserted central catheters (PICCs), or implantable ports (PORTs).

### 2.3. Clinical Endpoints

The primary clinical outcome under investigation was the first episode of unplanned removal of CVCs per patient, characterized as the early and unintended termination of a central venous access device (CVAD). This situation arises when the CVC is taken out accidentally by the patient or medical staff, or because of unexpected situations, prior to finishing the planned treatment course [7, 18]. To address multiple CVC insertion events within the same patient, only the initial CVC insertion event was analyzed. Unplanned CVC removal was systematically categorized into clinically relevant groups, namely: infection-related removal, occlusion, thrombosis, extravasation, and accidental removal. All variables were precisely defined, referring to the standards of infusion therapy from both the United States of America and China [19, 20]. In cases where no established definitions were available from these sources, definitions were devised by the authors (see Supporting Table S2 for detailed variable definitions).

### 2.4. Data Collection

For each enrolled patient, we systematically collected comprehensive baseline characteristics, including demographic data (age and sex), clinical information (primary diagnosis and comorbidities), therapeutic interventions (antibiotic use, parenteral nutrition, immunosuppressants such as glucocorticoids, and mechanical ventilation), and CVC-related parameters (insertion unit, operator, urgency of insertion, and indwelling duration in days). Detailed documentation of CVC removal, with particular attention to unplanned removals, was performed. The surveillance process commenced immediately upon CVC placement and continued until the earliest of the following events: 48 h post-CVC removal, discharge or transfer to another unit, or the patient's mortality. This systematic and continuous surveillance approach ensured that all relevant data were accurately and consistently collected for a comprehensive analysis. We followed each case until the catheter was removed, whether planned or unplanned, and documented the reasons for removal. To ensure data completeness, we implemented a comprehensive electronic data management system using a secure online platform with mandatory fields and real-time validation rules to prevent incomplete submissions. All center investigators received standardized training on data collection protocols, and each center appointed dedicated data managers to conduct secondary reviews. Our central team performed weekly audits of systematically sampled records, requiring resolution of any discrepancies within 48 h. This integrated approach combining automated completeness checks with manual verification by trained personnel at both institutional and central levels enabled immediate identification and correction of potential data gaps, resulting in a final analytic dataset with complete case reporting for all variables without missing values, and follow-up was complete for all eligible patients.

Between September 2023 and February 2024, all data were acquired exclusively through a web-based research electronic data platform, with no intermediate paper-based documentation. Initial implementation involved obtaining institutional commitments from 22 nursing managers across participating hospitals. Then, standardized training was provided to the designated nursing staff for data entry and submission, focusing on participant enrollment and data collection methods. Digital training materials and electronic informed consent documentation were distributed to facilitate this process. These trained personnel subsequently assumed primary responsibility for participant identification and comprehensive data compilation.

The same assessment methods were applied consistently across all participating sites and patient groups. We implemented a rigorous standardization protocol that included centralized training for all assessors using validated instructional materials; a comprehensive, standardized operating manual detailing all assessment procedures; and dual-level quality control measures consisting of both research team-based audits and site-based quality assurance checks to assure the quality and homogeneity of the assessment process.

We implemented a rigorous multi-faceted approach throughout our multicenter study to mitigate potential bias. Regarding selection bias, while our study was conducted across 22 tertiary hospitals, we ensured broad geographic representation by including ICUs from all seven major regions of China, enhancing the generalizability of our findings. We established strict, standardized eligibility criteria uniformly applied across all participating centers and implemented automated data linkage with hospital electronic records to minimize attrition bias. To control for measurement bias, we developed and executed a comprehensive quality assurance protocol that included centralized training of all assessors using validated instructional materials, a detailed operations manual with precisely defined outcome measures (e.g., unplanned CVC removal, CLABSI, occlusion), and a dual-layer quality control system featuring both on-site audits by nurse managers and remote electronic monitoring by coordinating researchers, complemented by monthly cross-center meetings to ensure data consistency and resolve any discrepancies. These methodical strategies have been used to maximize the robustness and reliability of the research findings.

### 2.5. Study Size

This study was designed as a multicenter prospective cohort study, enrolling patients with indwelling CVCs in the ICUs of 22 tertiary hospitals nationwide. The sample size calculation was based on the following two key parameters: First, considering the natural fluctuation of the number of patients admitted to ICUs in each center during the study period, we adopted the actual enrollment strategy; second, to ensure that the study was statistically valid, we set a minimum of 1000 CVC days of observational data to be accumulated at each center, taking into account the international standards for similar studies [7, 8]. Real-time surveillance of catheter-day accrual was implemented through integrated electronic medical record systems, with CVC days calculated using standardized methodology (daily enumeration of indwelling catheters at fixed timepoints) until protocol-specified observational endpoints were achieved, thereby optimizing both the precision of incidence estimates and the generalizability of findings across diverse ICU settings.

### 2.6. Statistical Methods

Statistical analysis was conducted using R software, Version 4.4.1. Medians and interquartile ranges (IQRs) were reported for continuous variables (age, duration of CVC days). In contrast, counts and proportions (%) were used to characterize categorical variables (e.g., gender, diagnosis, comorbidities). Besides, based on China's official legal and policy documents, the standardized age classification system categorizes the population into two distinct groups (< 60 vs. ≥ 60 years). Descriptive methods were employed to outline the characteristics of patients, CVCs, and the insertion procedures for both the entire cohort and individual CVCs. Reasons for CVC removal and unplanned CVC removal were summarized using counts and percentages with 95% confidence intervals (CIs) and incidence rates (IRs) per 1000 CVC days with 95% CIs. We have included proportion calculations with Wilson score intervals and Clopper–Pearson exact methods for rare events (the binom packages). IRs were calculated as events per 1000 CVC days, with 95% Poisson exact CIs (the epiR packages). Univariable Cox proportional hazards regression was performed to identify potential risk factors (*p* < 0.05 for inclusion). Variables meeting this threshold were entered into the multivariable Cox model for adjustment (retention criterion: *p* < 0.05). The results were presented as hazard ratios (HRs) with corresponding 95% CIs. The proportional hazards assumption was verified using Schoenfeld residuals (all *p* > 0.05). Besides unplanned CVC removal as the primary endpoint, all other termination events—including non-CVC-related death, planned removal per treatment protocol, and patient discharge/transfer—were right-censored in the primary analysis. To explore potential gender differences, we performed subgroup analyses stratified by gender and examined interaction effects between gender and key variables. Sensitivity analysis was conducted by excluding patients who underwent urgent CVC insertion to assess model robustness. A *p*-value below 0.05 was deemed to indicate statistical significance.

In this study, we employed a rigorous two-tiered approach to prevent missing data: First, through real-time electronic validation during data entry where the online platform enforced mandatory fields and flagged incompleteness for immediate correction; second, via systematic manual verification of all case records by trained personnel prior to analysis, with any identified gaps requiring resolution through direct follow-up with data collectors. These proactive quality control measures ensured complete case reporting, resulting in a final analytic dataset containing no missing values for any study variables.

## 3. Results

### 3.1. Patients and CVC Characteristics

**Figure 1:**
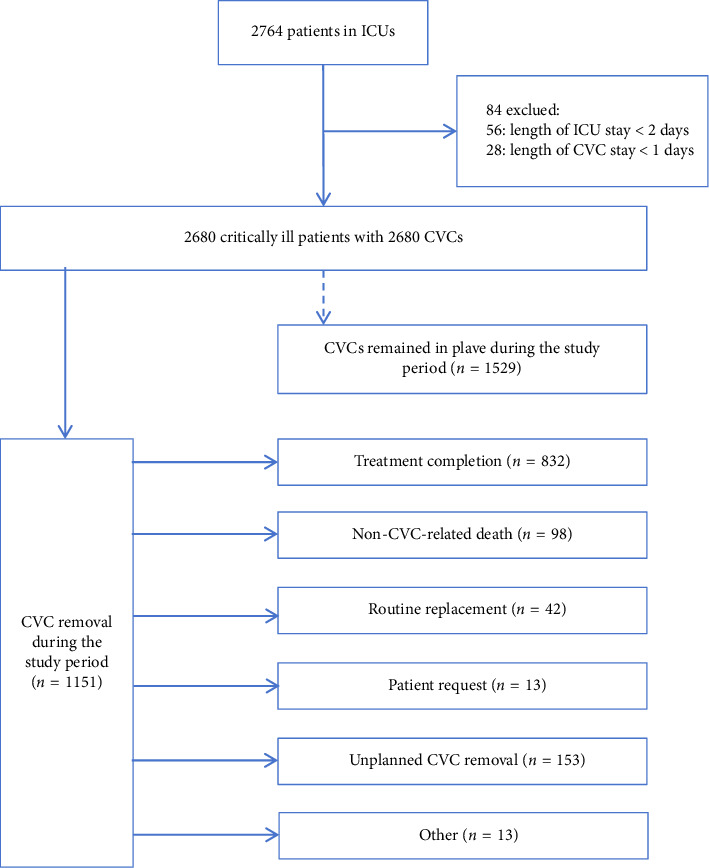
Flowchart of the study population, exclusion criteria, and study arms. ICU: intensive care unit; CVC: central venous catheter.

This prospective cohort study initially screened 2764 critically ill patients, with 84 exclusions applied following a priori protocol criteria (56 for ICU stays < 48 h and 28 for length of CVC days < 24 h), yielding an analytic cohort of 2680 patients. The standardized enrollment workflow is detailed in Figure 1. The final analytic dataset of 2680 patients contained no missing values for any study variables. All enrolled patients completed follow-up, with a median follow-up duration of 7 days [IQR: 5–12], ranging from a minimum 2 days to a maximum 131 days. The study cohort predominantly comprised older adults (median [IQR] age: 65 years [51–75]), with a male predominance (*n* = 1675, 62.5%). Age stratification revealed that 39.5% of participants were under 60 years, while the majority (60.5%) were aged 60 or older. Digestive disorders represented the leading cause of ICU admission, accounting for 26.8% of cases. In terms of comorbidities, one or more comorbidities were documented for 1629 patients (60.8%), 530 (19.8%) were diagnosed with diabetes, 999 (37.7%) had hypertension, 295 (11.0%) suffered from coronary heart disease, and 200 (7.5%) had stroke. Over half of the patients received parenteral nutrition (*n* = 1449, 54.1%) and mechanical ventilation (*n* = 1822, 68.0%), while one-quarter were administered immunosuppressive therapy (*n* = 670, 25.0%).

During the study period, a total of 2680 first-time CVC insertion events (one per patient) were included in the primary analysis. The majority of CVCs were inserted in ICUs (*n* = 1650, 61.6%) by intensive care physicians (*n* = 1691, 63.1%). CVCs were predominantly inserted at the internal jugular site (*n* = 1626, 60.7%), subclavian site (755, 28.2%), and femoral site (*n* = 218, 8.1%). 438 (16.3%) CVCs were inserted urgently, and the average duration of CVC days was 6 days [IQR: 4–10], ranging from a minimum 1 day to a maximum 129 days. More details are summarized in Table 1.

### 3.2. Occurrence of CVC Removal

Among 2680 successfully inserted CVCs, 1151 (42.95%) were removed during the study period, while 1529 (57.05%) remained in place. Of the removed catheters, the most common reason was planned removal following completion of the prescribed therapy (*n* = 832, 31.04%). Other planned removal reasons included patient death unrelated to CVC use (*n* = 98, 3.66%), routine replacement (*n* = 42, 1.57%), patient request (*n* = 13, 0.48%), and other physician-documented indications (*n* = 13, 0.49%). The remaining 153 removals (5.71%) were classified as premature due to complications or other unplanned events (see Table 2 for more details).

### 3.3. Occurrence of Unplanned CVC Removal

Of the 2680 CVCs inserted during the study period, 153 CVCs were removed prematurely (5.71%; 95% CI: 4.84–6.68), at an IR of 6.49 per 1000 CVC days (95% CI: 5.50–7.61). Infection-related complications accounted for the majority of premature removals (*n* = 24, 4.63%; 95% CI: 3.85–5.50; 5.26 per 1000 CVC days), including 20 confirmed central line-associated bloodstream infection (CLABSI) cases (0.75%, 95% CI: 0.46–1.15; 0.85 per 1000 CVC days) and 104 suspected CLABSI cases (3.88%, 95% CI: 3.17–4.68; 4.41 per 1000 CVC days). In total, 25 CVCs were diagnosed with CLABSI over the study period. Of these, 20 were prematurely removed due to CLABSI, while 4 were removed for other reasons (1 after treatment completion, 1 following non-CVC-related deaths, 1 due to occlusion, and 1 from extravasation). Notably, 1 CLABSI-diagnosed CVC remained in place at the end of the study period. Other significant reasons for unplanned CVC removal included CVC occlusion (*n* = 20, 0.75%; 95% CI: 0.46–1.15; 0.85 per 1000 CVC days), thrombosis (*n* = 3, 0.11%; 95% CI: 0.02–0.33; 0.13 per 1000 CVC days), extravasation (*n* = 5, 0.19%; 95% CI: 0.06–0.44; 0.21 per 1000 CVC days), and accidental removal (*n* = 1, 0.04%; 95% CI: 0.00–0.21; 0.04 per 1000 CVC days; details are listed in Table 3).

### 3.4. Risk Factors for Unplanned CVC Removal

Univariable Cox proportional hazards regression was performed to identify potential risk factors (*p* < 0.05 for inclusion). Variables meeting this threshold were entered into the multivariable Cox model for adjustment (retention criterion: *p* < 0.05), as detailed in Table 4. In comparison to the reference group, multiple variables significantly increased the likelihood of unplanned CVC removal. Males had a 2.04-fold increased risk (HR, 2.04; 95% CI: 1.40–2.99; *p* < 0.001). Patients diagnosed with neurological disorders also demonstrated a significantly higher risk (HR, 2.41; 95% CI: 1.50–3.86; *p* < 0.001). Mechanical ventilation contributed to a 71% greater likelihood (HR, 1.71; 95% CI: 1.09–2.70; *p*=0.02). Conversely, urgent insertion procedures were found to be protective against unplanned CVC removal, reducing the risk by 48% (HR, 0.52; 95% CI: 0.29–0.92; *p*=0.02). The proportional hazards assumption was not violated (global *p*=0.45), indicating the appropriateness of the Cox model (Supporting Figure S1).

Subgroup analyses by gender revealed distinct risk factor patterns for unplanned CVC removal. Among male patients, diagnosis with neurological disorders demonstrated a particularly strong association (HR, 2.31; 95% CI: 1.40–3.81; *p*=0.001), while urgent CVC insertion showed a protective effect (HR, 0.41; 95% CI: 0.21–0.82; *p*=0.01). In contrast, female patients exhibited nonsignificant trends for these factors (neurological disorders: HR, 1.73, 95% CI: 0.56–5.31, *p*=0.34; urgent insertion: HR, 0.72, 95% CI: 0.25–2.06, *p*=0.54). Mechanical ventilation showed borderline significance in males (HR, 1.61; 95% CI: 0.98–2.64; *p*=0.06) and a stronger but nonsignificant association in females (HR, 2.38; 95% CI: 0.83–6.81; *p*=0.11). No significant interactions were found between gender and diagnosis, mechanical ventilation, or urgent insertion (all *p* > 0.05; Supporting Table S3).

In the sensitivity analysis excluding patients who underwent urgent CVC insertion, multivariable Cox regression showed that male gender (HR, 2.18; 95% CI: 1.45–3.26; *p*=0.002), neurological disorders (HR, 2.66; 95% CI: 1.61–4.40; *p*=0.001), and mechanical ventilation (HR, 1.84; 95% CI: 1.13–2.98; *p*=0.01) remained significantly associated with unplanned CVC removal, confirming the robustness of our main findings (Supporting Table S4).

## 4. Discussion

The study cohort comprised a total of 2680 first-time CVC insertion events (one per patient), with removal occurring in 42.95% of CVCs. The predominant reason for CVC removal was treatment completion, accounting for 31.04% of all removal cases. This finding aligns with the fundamental purpose of CVC placement, which is to support specific therapeutic interventions until their intended course is fulfilled. Curtis and colleagues [14] documented approximately double the removal rate for CVADs relative to our observations. Despite this difference in the magnitude of removal risk, the predominant reason for catheter removal was consistent between the two studies. This consistency across different studies highlights the universality of treatment-related factors in determining the duration of CVC use. It also implies that, regardless of the variation in removal risk, the underlying clinical decision-making process regarding catheter removal, primarily based on the completion of treatment, remains relatively stable. Moreover, the observed variation likely stems from differences in patient characteristics, institutional catheter management practices, or monitoring approaches affecting utilization patterns.

Our study found that 5.71% of CVCs were removed prematurely. This rate is notably lower compared to previous research findings. For instance, in individuals suffering from hematological malignancies, the reported risk of unplanned removal of CVADs reached as much as 42% [14]. Among children with hematologic-oncologic disorders, the risk of unplanned CVC removal varied between 20% and 25% [16]. A single-institutional study from the United Kingdom documented an unplanned CVAD removal risk of 38.2% among children [11]. Among oncology patients, 26% of PICCs and 18% of PORTs were removed prematurely [21]. Our data align closely with pediatric intensive care unit (PICU) reports documenting 8.9% premature removal rates [7]. Regarding the causes of unplanned CVC removal, our study indicated that infection was the primary factor. This finding aligns with the reports by Shimizu [7] and Curtis [14]. In contrast, Garonzi [16] and Akhtar [21] reported mechanical complications as the main cause of unplanned catheter removal. These discrepancies highlight the diverse clinical scenarios and patient characteristics across different studies, underscoring the complexity of unplanned CVC removal and the need for context-specific preventive strategies. The variation in the predominant causes of unplanned catheter removal also implies that risk assessment and management strategies should be adapted to different patient groups, considering both the nature of the underlying conditions and the features of the inserted catheters.

Univariable and multivariable Cox regression analyses identified several significant risk factors for unplanned CVC removal. Males, patients undergoing mechanical ventilation, exhibited an elevated risk of unplanned CVC removal. These findings align with previous research, which has consistently associated male gender [22, 23] and mechanical ventilation [24, 25] with an increased likelihood of catheter-related complications, ultimately predisposing patients to unplanned CVC removal. Notably, our study revealed that gender modifies the effect of certain risk factors on unplanned removal. In male patients, neurological disorders were a major risk factor, which may be attributed to multiple factors. Neurological disorders often lead to varying degrees of physiological dysfunction and immunosuppression, compromising the body's ability to tolerate and maintain the catheter. Additionally, these patients frequently experience altered states of consciousness, such as confusion, delirium, or decreased awareness, which are well-recognized contributors to unplanned catheter removal [9, 26]. Contrary to previous reports, our study found that urgent CVC insertion procedures were protective against unplanned CVC removal. In contrast, Curtis [14] reported that urgent CVC insertion increased the risk of unplanned CVC removal. Conventional wisdom suggests urgent insertions carry greater complication risks, prompting guidelines to advocate early removal when sterility is questionable [27, 28]. However, recent advancements in insertion technologies [29], disinfection materials [30], and the implementation of standardized preventive interventions for CVC management may have reduced the potential for catheter-related complications and occurrence of unplanned CVC removal. Population heterogeneity, procedural variations, and maintenance protocols likely contribute to these conflicting results. These divergent results underscore the need for further research to elucidate the complex relationship between CVC insertion urgency and unplanned removal risk.

Beyond patient-related and CVC-related factors, healthcare provider-related factors play a crucial role in unplanned CVC removal. Curtis [14] reported that insertion technology is linked to the risk of unplanned CVAD removal. Shimizu [7] further supported that technical factors, including tip positioning accuracy and ultrasound utilization, decrease unplanned removal incidents. Balmforth [9] emphasized that training and assessment in suturing techniques, structured documentation of catheter insertion, and systematic patient reviews during rounding are effective measures to prevent unplanned catheter removal. Our prior investigations evaluated operating room nurses' CLABSI prevention competencies. Findings revealed moderate levels of knowledge, attitude, and practice among nurses regarding CLABSI prevention. Notably, nurses who had participated in CLABSI-specific training achieved significantly higher scores in all dimensions [31]. We also investigated ICU nurses' knowledge, attitude, and practice regarding CLABSI prevention. The findings revealed that Chinese ICU nurses had inadequate levels of knowledge and attitude toward CLABSI prevention [32]. Additionally, our research on evidence-based practices and adherence rates in CVC maintenance among ICU nurses indicated that ICU nurses' adherence to the CVC maintenance bundle was less than satisfactory. The low adherence to certain items was seemingly attributed to factors such as a lack of interdepartmental collaboration, insufficient training, and heavy workloads [33]. Therefore, targeted educational programs should be held to boost healthcare providers' knowledge and skills in preventing catheter-related complications and unplanned catheter removal. Multidisciplinary collaboration among physicians, nurses, and hospital administrators is needed. Standardized protocols should be developed for real-time decision support and to minimize practice variability. Moreover, continuous monitoring and feedback to clinical teams about complication and unplanned catheter removal rates are indispensable for timely practice adjustments and overall quality improvement in catheter-related care.

Our study's strengths include its large multicenter sample, strict eligibility criteria, and proactive data collection using an online system. However, the study has limitations. First, as its observational design, residual confounding cannot be ruled out entirely. This study was conducted in 22 tertiary hospitals, which are among the best in the country in terms of medical standards and conditions of care, and may not reflect the true situation in all healthcare facilities across the country, underestimating the actual occurrence of unplanned CVC removal. Furthermore, only patients with indwelling CVC alone were included in this study, which may have led to the exclusion of patients with concurrent catheters, such as CVC and PICC, simultaneously, also underestimating the actual occurrence of unplanned CVC removal. Second, our study focused solely on patient and CVC-related risk factors, not considering other factors like health provider, practice context, or healthcare system-related factors, which may be associated with catheter-related complications and unplanned catheter removal. Third, while this multicenter cohort study employed standardized assessment protocols across 22 hospitals (50 ICUs) and included a broad patient population (neonates to elderly with multiple comorbidities), heterogeneity in CVC insertion and maintenance practices across sites may limit the generalizability of findings. Caution should be exercised when extrapolating these results to non-ICU settings or international contexts, as institution-specific factors (e.g., staffing ratios and procedural protocols) and healthcare system characteristics may significantly influence outcomes.

## 5. Conclusion

This multicenter cohort study, implemented in 22 large-scale tertiary public hospitals in China, reported a relatively low incidence of unplanned CVC removal. Among critically ill individuals, catheter-related infection emerged as the primary cause of unplanned CVC removal. Specifically, male patients, those diagnosed with neurological disorders, and patients undergoing mechanical ventilation were found to have an increased likelihood of unplanned CVC removal. In contrast, urgent CVC insertion procedures were associated with a decreased risk of unplanned CVC removal.

Nevertheless, certain uncertainties remain. These uncertainties stem from the inherent selection bias inherent in the observational study design. To address these issues and enhance the quality of care related to unplanned CVC removal, it is advisable to initiate quality improvement projects. Such projects could involve strengthening personalized education and training programs, promoting interdisciplinary cooperation, formulating standardized work processes, and establishing an efficient monitoring mechanism. It is anticipated that these actions will help decrease the rate of unplanned CVC removal in ICUs.

## Figures and Tables

**Table 1:** Patient and central venous catheter characteristics.

	*n* (%)
*Patient characteristics*	
Male	1675 (62.5)
Age (years) (median, [IQR])	65.00 [51.00, 75.00]
< 60	1058 (39.5)
≥ 60	1622 (60.5)
Diagnosis	
Digestive disorders	717 (26.8)
Circulatory disorders	577 (21.5)
Respiratory disorders	439 (16.4)
Neurological disorders	398 (14.9)
Others	549 (20.4)
Comorbidities	
Diabetes	530 (19.8)
Hypertension	999 (37.3)
Coronary heart disease	295 (11.0)
Stroke	200 (7.5)
Parenteral nutrition	1449 (54.1)
Use of immunosuppressants	670 (25.0)
Mechanical ventilation	1822 (68.0)

*CVC characteristics*	
Place for CVC insertion	
Non-ICU	1030 (38.4)
ICU	1650 (61.6)
Operator for CVC insertion	
Anesthetists	989 (36.9)
Physicians	1691 (63.1)
Insertion site	
Subclavian	755 (28.2)
Internal jugular	1626 (60.7)
Femoral	218 (8.1)
Other	81 (3.0)
Urgent CVC insertion	438 (16.3)
Duration of CVC days (days) (median, [IQR])	6.00 [4.00, 10.00]

*Note:* Categorical variables are expressed as number of patients (%); continuous variables are expressed as median (IQR).

Abbreviations: CVC = central venous catheter, ICU = intensive care unit, IQR = interquartile range (75th quartile minus 25th quartile).

**Table 2:** Summary of central venous catheter removal (*n* = 2943, 23,582 CVC days).

Event	Episodes	Proportion of CVCs (%)	95% CI	Incidence rate per 1000 CVC days	95% CI
CVC removal, total	1151	42.95	40.53–45.42	48.80	46.05–51.65
Treatment completion	832	31.04	29.03–33.12	35.28	32.89–37.78
Non-CVC-related death	98	3.66	2.97–4.43	4.16	3.38–5.06
Routine replacement	42	1.57	1.13–2.10	1.78	1.28–2.40
Patient request	13	0.48	0.26–0.83	0.55	0.29–0.94
Unplanned CVC removal	153	5.71	4.84–6.68	6.49	5.50–7.61
Other	13	0.49	0.26–0.83	0.55	0.29–0.94

Abbreviations: CI = confidence interval, CVC = central venous catheter.

**Table 3:** Summary of unplanned central venous catheter removal (*n* = 2943, 23,582 CVC days).

Event	Episodes	Proportion of CVCs (%)	95% CI	Incidence rate per 1000 CVC days	95% CI
Unplanned CVC removal, total	153	5.71	4.84–6.68	6.49	5.50–7.61
Infection-related removal	124	4.63	3.85–5.50	5.26	4.37–6.26
CLABSI^∗^	20	0.75	0.46–1.15	0.85	0.52–1.31
Suspected CLABSI	104	3.88	3.17–4.68	4.41	3.60–5.34
Occlusion	20	0.75	0.46–1.15	0.85	0.52–1.31
Thrombosis	3	0.11	0.02–0.33	0.13	0.03–0.37
Extravasation	5	0.19	0.06–0.44	0.21	0.07–0.50
Accidental removal	1	0.04	0.00–0.21	0.04	0.00–0.24

Abbreviations: CI = confidence interval, CLABSI = central line-associated bloodstream infection, CVC = central venous catheter.

^∗^In total, 25 CVCs were diagnosed with CLABSI over the study period. Of these, 20 were prematurely removed due to CLABSI, while 4 were removed for other reasons (1 after treatment completion, 1 following non-CVC-related deaths, 1 due to occlusion, and 1 from extravasation). Notably, 1 CLABSI-diagnosed CVC remained in place at the end of the study period.

**Table 4:** Risk factors for unplanned CVC removal.

Variables	Univariable analysis		Multivariable analysis	
	HR (95% CI)	*p* value	HR (95% CI)	*p* value
Gender				
Female	Reference		Reference	
Male	2.02 (1.38–2.95)	**< 0.001**	2.04 (1.40–2.99)	**< 0.001**
Age				
< 60 years	Reference			
≥ 60 years	0.78 (0.57–1.07)	0.12		
Diagnosis				
Digestive disorders	Reference		Reference	
Circulatory disorders	0.88 (0.51–1.52)	0.65	1.02 (0.58–1.79)	0.95
Neurological disorders	2.27 (1.44–3.57)	**< 0.001**	2.41 (1.50–3.86)	**< 0.001**
Respiratory disorders	1.03 (0.61–1.74)	0.91	1.08 (0.62–1.88)	0.79
Other	1.07 (0.63–1.82)	0.80	1.30 (0.75–2.25)	0.35
Comorbidities				
Diabetes (yes)	0.72 (0.47–1.10)	0.13		
Hypertension (yes)	0.86 (0.62–1.21)	0.39		
Coronary heart disease (yes)	0.69 (0.39–1.22)	0.20		
Stroke (yes)	0.77 (0.42–1.42)	0.40		
Parenteral nutrition (yes)	0.94 (0.68–1.30)	0.72		
Use of immunosuppressants (yes)	1.43 (1.03–1.99)	**0.03**	1.23 (0.87–1.72)	0.25
Mechanical ventilation (yes)	1.73 (1.11–2.70)	**0.02**	1.71 (1.09–2.70)	**0.02**
Unit for CVC insertion				
ICU	Reference			
Non-ICU	1.37 (0.99–1.90)	0.06		
Insertion site				
Subclavian site	Reference		Reference	
Femoral site	1.60 (0.90–2.82)	0.11	1.27 (0.71–2.29)	0.42
Internal jugular site	1.46 (0.98–2.19)	0.06	1.37 (0.89–2.12)	0.15
Other	2.27 (1.08–4.76)	**0.03**	2.32 (0.43–5.06)	0.35
Operator for CVC insertion				
Physicians	Reference			
Anesthetists	1.52 (1.10–2.10)	**0.01**	1.34 (0.92–1.93)	0.12
Urgent CVC insertion (yes)	0.45 (0.25–0.79)	**0.006**	0.52 (0.29–0.92)	**0.02**

*Note:p* value was calculated from the Cox proportional hazards model. A univariable Cox proportional hazards regression model was performed to screen potential variables. Variables with *p* < 0.05 in univariable analysis were entered into a multivariable Cox regression model for adjustment. The bolded values indicate statistical significance at the level of *p* < 0.05.

Abbreviations: CVC = central venous catheter, HR = hazard ratios, IC = confidence intervals, ICU = intensive care unit.

## Data Availability

The data that support the findings of this study are available on request from the corresponding author. The data are not publicly available due to privacy or ethical restrictions.

## Supporting Information

Figure S1: Proportional hazards assumption check. Dots represent scaled Schoenfeld residuals with local regression (loess) smoothing lines (red curves). The dashed horizontal line at *y* = 0 indicates the reference where hazards are perfectly proportional. Formal test results for each covariate: gender (*χ*^2^ = 0.16, *p*=0.69), diagnosis (*χ*^2^ = 6.72, *p*=0.15), use of immunosuppressants (*χ*^2^ = 2.49, *p*=0.11), mechanical ventilation (*χ*^2^ = 0.74, *p*=0.39), site (*χ*^2^ = 0.76, *p*=0.86), personnel (*χ*^2^ = 1.51, *p*=0.22), and urgent insertion (*χ*^2^ = 0.33, *p*=0.573). Global test: *χ*^2^ = 11.96, *p*=0.45. All *p* values > 0.05 indicate that the proportional hazards assumption is satisfied for all covariates. Table S1: STROBE Statement—Checklist of items that should be included in reports of cohort studies. Table S2: Definitions of variables related to unplanned CVC removal. Table S3: Subgroup analysis of risk factors for unplanned CVC removal by gender. Table S4: Sensitivity analysis of risk factors for unplanned removal (excluding patients with urgent CVC insertion).
