# Supplementary material for: Occurrence and Risk Factors for Unplanned Central Venous Catheter Removal in Critically Ill Patients: A Multicenter Cohort Study
Source: Nurs Res Pract. 2025 Sep 4;2025:7640284. doi: 10.1155/nrp/7640284 (PMC12425627; doi:10.1155/nrp/7640284)
Supplement: Supporting Information — Additional supporting information can be found online in the Supporting Information section. [file 7640284.f1.docx]

**Supplementary file:**

**Table S1 STROBE Statement—Checklist of items that should be included in reports of cohort studies**

**Table S2 Definitions of variables related to unplanned CVC removal**

**Table S3 Subgroup Analysis of Risk Factors for Unplanned CVC removal by Gender**

**Table S4 Sensitivity Analysis of Risk Factors for Unplanned Removal (Excluding patients with urgent CVC insertion)**

**Figure S1 Proportional hazards assumption check**

**Table S1**

STROBE Statement—Checklist of items that should be included in reports of ***cohort studies***

|  | Item No | Recommendation | Page No |
| --- | --- | --- | --- |
| **Title and abstract** | 1 | (*a*) Indicate the study’s design with a commonly used term in the title or the abstract | 1 |
|  |  | (*b*) Provide in the abstract an informative and balanced summary of what was done and what was found | 2-3 |
| Introduction | | | |
| Background/rationale | 2 | Explain the scientific background and rationale for the investigation being reported | 3-4 |
| Objectives | 3 | State specific objectives, including any prespecified hypotheses | 4 |
| Methods | | | |
| Study design | 4 | Present key elements of study design early in the paper | 5 |
| Setting | 5 | Describe the setting, locations, and relevant dates, including periods of recruitment, exposure, follow-up, and data collection | 5-8 |
| Participants | 6 | (*a*) Give the eligibility criteria, and the sources and methods of selection of participants. Describe methods of follow-up | 5-8 |
|  |  | (*b*) For matched studies, give matching criteria and number of exposed and unexposed | NA |
| Variables | 7 | Clearly define all outcomes, exposures, predictors, potential confounders, and effect modifiers. Give diagnostic criteria, if applicable | 5-6 |
| Data sources/ measurement | 8* | For each variable of interest, give sources of data and details of methods of assessment (measurement). Describe comparability of assessment methods if there is more than one group | 6-8 |
| Bias | 9 | Describe any efforts to address potential sources of bias | 7-8 |
| Study size | 10 | Explain how the study size was arrived at | 8 |
| Quantitative variables | 11 | Explain how quantitative variables were handled in the analyses. If applicable, describe which groupings were chosen and why | 8-9 |
| Statistical methods | 12 | (*a*) Describe all statistical methods, including those used to control for confounding | 9-10 |
|  |  | (*b*) Describe any methods used to examine subgroups and interactions | 9-10 |
|  |  | (*c*) Explain how missing data were addressed | 10 |
|  |  | (*d*) If applicable, explain how loss to follow-up was addressed | 10 |
|  |  | (*e*) Describe any sensitivity analyses | 10 |
| Results | | |  |
| Participants | 13* | (a) Report numbers of individuals at each stage of study—eg numbers potentially eligible, examined for eligibility, confirmed eligible, included in the study, completing follow-up, and analysed | 10 |
|  |  | (b) Give reasons for non-participation at each stage | 10 |
|  |  | (c) Consider use of a flow diagram | Figure1 |
| Descriptive data | 14* | (a) Give characteristics of study participants (eg demographic, clinical, social) and information on exposures and potential confounders | 10-11 |
|  |  | (b) Indicate number of participants with missing data for each variable of interest | 10 |
|  |  | (c) Summarise follow-up time (eg, average and total amount) | 10 |
| Outcome data | 15* | Report numbers of outcome events or summary measures over time | 11-13 |

| Main results | 16 | (*a*) Give unadjusted estimates and, if applicable, confounder-adjusted estimates and their precision (eg, 95% confidence interval). Make clear which confounders were adjusted for and why they were included | 12-13 |
| --- | --- | --- | --- |
|  |  | (*b*) Report category boundaries when continuous variables were categorized | 10 |
|  |  | (*c*) If relevant, consider translating estimates of relative risk into absolute risk for a meaningful time period | NA |
| Other analyses | 17 | Report other analyses done—eg analyses of subgroups and interactions, and sensitivity analyses | 13 |
| Discussion | | | |
| Key results | 18 | Summarise key results with reference to study objectives | 13-17 |
| Limitations | 19 | Discuss limitations of the study, taking into account sources of potential bias or imprecision. Discuss both direction and magnitude of any potential bias | 17 |
| Interpretation | 20 | Give a cautious overall interpretation of results considering objectives, limitations, multiplicity of analyses, results from similar studies, and other relevant evidence | 13-17 |
| Generalisability | 21 | Discuss the generalisability (external validity) of the study results | 17 |
| Other information | | | |
| Funding | 22 | Give the source of funding and the role of the funders for the present study and, if applicable, for the original study on which the present article is based | 19 |

*Give information separately for exposed and unexposed groups.

**Note:** An Explanation and Elaboration article discusses each checklist item and gives methodological background and published examples of transparent reporting. The STROBE checklist is best used in conjunction with this article (freely available on the Web sites of PLoS Medicine at http://www.plosmedicine.org/, Annals of Internal Medicine at http://www.annals.org/, and Epidemiology at http://www.epidem.com/). Information on the STROBE Initiative is available at http://www.strobe-statement.org.

**Table S2 Definitions of variables related to unplanned CVC removal**

| Variable | Definition | Reference |
| --- | --- | --- |
| Unplanned CVC removal | the premature and involuntary discontinuation of a central venous access device. This occurs when the CVC is removed either inadvertently by the patient or healthcare provider, or due to unforeseen circumstances, before completion of the intended course of therapy | (Shimizu et al., 2020; Curtis et al., 2024) |
| CLABSI | Central line-associated bloodstream infections (CLABSIs) refer to infections that are laboratory-confirmed in patients with a CVC for over 48 hours prior to blood culture collection, assuming no other source of bacterial or fungal infection is detected. CLABSI is typically used as a term for clinical surveillance and data reporting. The incidence of CLABSI is determined by the number of CLABSI cases recorded per 1000 CVC days. | (United States Centers for Disease Control and Prevention, 2024; National Health Commission of the People's Republic of China, 2017) |
| Occlusion | Mechanical occlusion: internal or external problems with the catheter. They can be the result of issues such as catheter or tubing kinks, CVC dislodgement or tip migration, a clogged cap/ needle-free connector or filter, or incorrect placement of a non-coring needle in a CVC  Chemical occlusions: medication or medication precipitate and can specifically be the result of precipitate from the mixing of incompatible solutions and/or medications or lipid residue.  thrombotic occlusions: CVCs are intraluminal thrombus, fibrin tail or flap, fibrin sheath or sleeve, and mural thrombus. | (Broadhurst et al., 2019) |
| Thrombosis | initiated as an inflammatory response to vessel wall injury and appears as an anechoic or hypoechoic image on ultrasonic evaluation, partially or fully occluding the vessel lumen. | (Nickel et al., 2024; National Health Commission of the People's Republic of China, 2023) |
| Extravasation | During intravenous therapy, the medication enters the surrounding tissues outside the lumen of the vein. During intravenous infusion, any type of swelling, abnormal sensation (coldness, numbness, etc.), pain, burning sensation, etc. found or complained by the patient at and around the puncture site, at the tip of the catheter, or throughout the venous route should be considered as oozing or extravasation. | (Nickel et al., 2024; National Health Commission of the People's Republic of China, 2023) |
| Accidental removal | any unintended CVC removal occurring before completion of prescribed therapy, regardless of causative agent (patient, nursing staff, or other healthcare personnel). | (Tran et al., 2023) |

**Reference:**

Shimizu, Y., Hatachi, T., Takeshita, J., Inata, Y., Kyogoku, M., Aoki, Y., Taniguchi, M., Kawamura, A., Okumura, J., & Takeuchi, M. (2020). Occurrence and Risk Factors for Unplanned Catheter Removal in a PICU: Central Venous Catheters Versus Peripherally Inserted Central Venous Catheters. Pediatr Crit Care Med, 21(9), e635-e642.

Curtis, K., Gough, K., Krishnasamy, M., Tarasenko, E., Hill, G., & Keogh, S. (2024). Central venous access device terminologies, complications, and reason for removal in oncology: a scoping review. BMC Cancer, 24(1), 498.

United States Centers for Disease Control and Prevention. (2024). 2022 National and State Healthcare-Associated Infections Progress Report.

National Health Commission of the People's Republic of China. (2017). Regulation for prevention and control of healthcare associated infection in intensive care unit. Chinese Journal of Infection Control, 16(02), 191-194.

Broadhurst, D., Cernusca, C., Cook, C., Hill, J., Naayer, K., Paquet, F., & Raynak, A. (2019). CVAA Occlusion Management Guideline for Central Venous Access Devices (CVADs) Vascular Access, 13(2019 Second Edition).

Nickel, B., Gorski, L., Kleidon, T., Kyes, A., DeVries, M., Keogh, S., Meyer, B., Sarver, M. J., Crickman, R., Ong, J., Clare, S., & Hagle, M. E. (2024). Infusion Therapy Standards of Practice, 9th Edition. Journal of Infusion Nursing, 47(1S), S1-S285.

National Health Commission of the People's Republic of China. (2023). Nursing practice standard for intravenous therapy.

Tran, A. T., Rizk, E., Aryal, D. K., Soto, F. J., & Swan, J. T. (2023). Incidence of Midline Catheter Complications Among Hospitalized Patients. J Infus Nurs, 46(1), 28-35.

**Table S3 Subgroup Analysis of Risk Factors for Unplanned CVC removal by Gender**

| Variable | Male | | Female | | Interaction p-value |
| --- | --- | --- | --- | --- | --- |
|  | HR (95% CI) | p-value | HR (95% CI) | p-value |  |
| Diagnosis-  Neurological disorders | 2.31 (1.40–3.81) | **0.001** | 1.73 (0.56–5.31) | 0.34 | 0.66 |
| Mechanical ventilation | 1.61 (0.98–2.64) | 0.06 | 2.38 (0.83–6.81) | 0.11 | 0.46 |
| Urgent CVC insertion | 0.41 (0.21–0.82) | **0.01** | 0.72 (0.25–2.06) | 0.54 | 0.34 |

Note: This table presents the results of multivariable Cox regression analyses stratified by gender. Hazard ratios (HR), 95% confidence intervals (CI), and p-values are shown for each variable. No significant interactions between gender and other variables were observed (p >0.05). Bold values indicate statistical significance at p < 0.05.

**Table S4 Sensitivity Analysis of Risk Factors for Unplanned Removal (Excluding patients with urgent CVC insertion)**

| Variables | HR (95% CI) | *P*-value |
| --- | --- | --- |
| Gender |  |  |
| Female | reference |  |
| Male | 2.18（1.45–3.26） | **0.002** |
| Diagnosis |  |  |
| Digestive disorders | reference |  |
| Circulatory disorders | 1.23（0.69–2.22） | 0.48 |
| Neurological disorders | 2.66（1.61–4.40） | **0.001** |
| Respiratory disorders | 1.24（0.68–2.25） | 0.48 |
| Other | 1.31（0.71–2.41） | 0.39 |
| Use of immunosuppressants (yes) | 1.22（0.85–1.74） | 0.28 |
| Mechanical ventilation (yes) | 1.84（1.13–2.98） | **0.01** |
| Insertion site |  |  |
| Subclavian site | reference |  |
| Femoral site | 1.27 (0.70-2.32) | 0.43 |
| Internal jugular site | 1.24 (0.79-1.94) | 0.36 |
| Other | 2.00 (0.88-4.56) | 0.10 |
| Operator for CVC insertion |  |  |
| Physicians | reference |  |
| Anesthetists | 1.37 (0.93-2.01) | 0.11 |

Note: Multivariable Cox regression analysis was performed excluding patients with urgent CVC insertion. Hazard ratios (HR), 95% confidence intervals (CI), and p-values are shown. Bold values indicate statistical significance at p < 0.05.

**Figure S1 Proportional hazards assumption check**

| 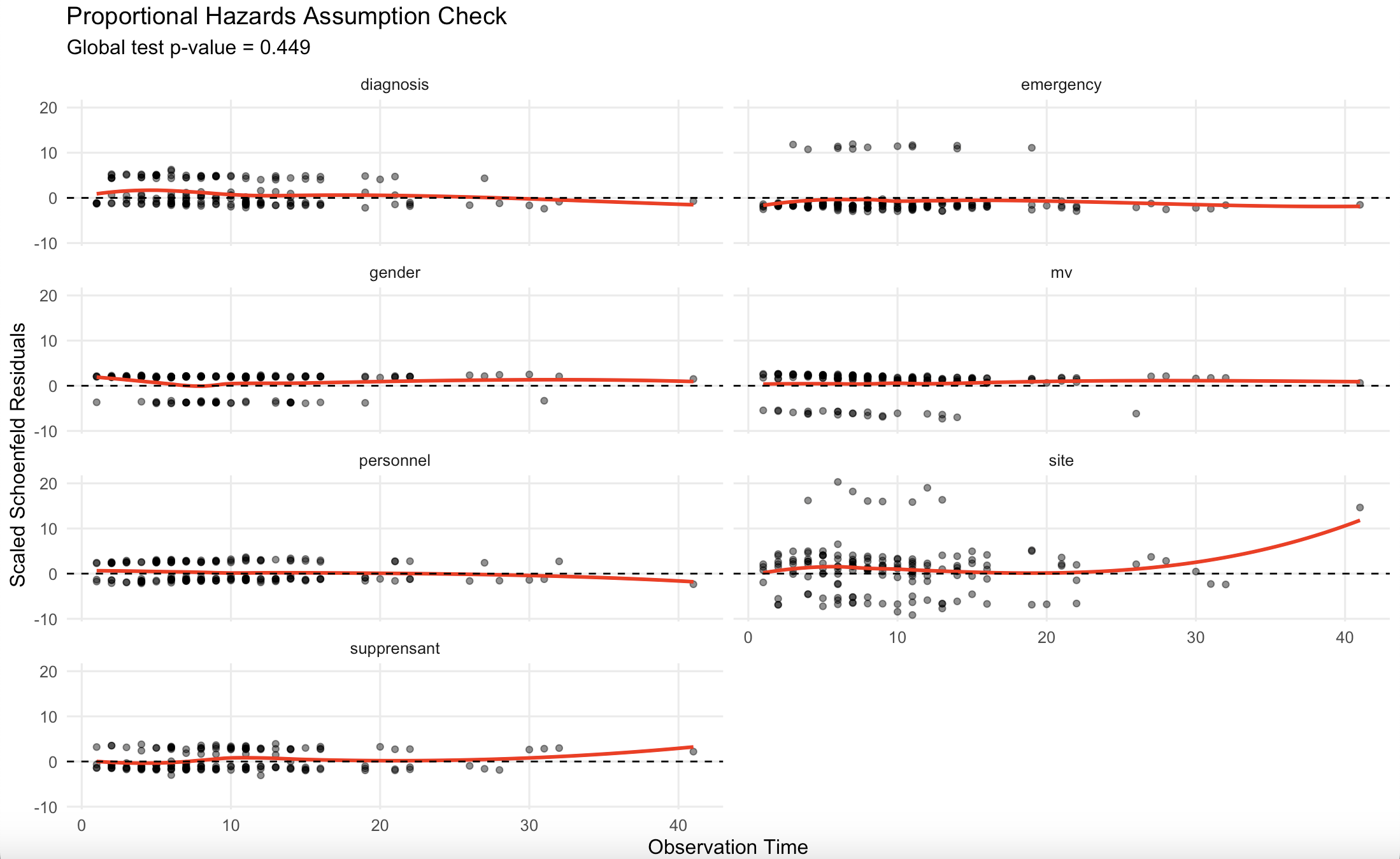 | |
| --- | --- |
| Note: Dots represent scaled Schoenfeld residuals with local regression (loess) smoothing lines (red curves). The dashed horizontal line at y=0 indicates the reference where hazards are perfectly proportional. Formal test results for each covariate: gender (χ²=0.16, p=0.69), diagnosis (χ²=6.72, p=0.15), use of immunosuppressants (χ²=2.49, p=0.11), mechanical ventilation (χ²=0.74, p=0.39), site (χ²=0.76, p=0.86) , personnel (χ²=1.51, p=0.22), urgent insertion (χ²=0.33, p=0.573). Global test: χ²=11.96, p=0.45. All p-values >0.05 indicate that the proportional hazards assumption is satisfied for all covariates. |  |
